# Diminished Returns of Educational Attainment on Welfare Receipt of American Indian/Alaska Native People: National Health Interview Survey (NHIS) 2023

**DOI:** 10.31586/ojp.2025.1149

**Published:** 2025-02-10

**Authors:** Shervin Assari, Amanda Sonnega, Hossein Zare

**Affiliations:** 1 Department of Internal Medicine, Charles R. Drew University of Medicine and Science, Los Angeles, CA, United States; 2 Department of Family Medicine, Charles R. Drew University of Medicine and Science, Los Angeles, CA, United States; 3 Department of Urban Public Health, Charles R. Drew University of Medicine and Science, Los Angeles, CA, United States; 4 Marginalization-Related Diminished Returns (MDRs) Center, Los Angeles, CA, United States; 5 Institute for Social Research, University of Michigan, Ann Arbor, MI, United States; 6 Department of Health Policy and Management, Johns Hopkins Bloomberg School of Public Health, Baltimore, MD, United States; 7 School of Business, University of Maryland Global Campus (UMGC), Adelphi, MD, United States

**Keywords:** Educational Attainment, Welfare Reliance, Racial Disparities, Socioeconomic Benefits, American Indian/Alaska Native

## Abstract

**Background:**

Educational attainment is generally associated with reduced reliance on Social Security and disability benefits; however, the Minorities’ Diminished Returns (MDRs) theory suggests that the socioeconomic benefits of education are weaker for minoritized populations. This study investigates the relationship between educational attainment and welfare receipt among American Indian/Alaska Native (AIAN) and White adults in the United States.

**Objective:**

Using the MDRs framework, we analyzed data from the National Health Interview Survey (NHIS) 2023 to examine how educational attainment impacts welfare receipt among AIAN and White adults.

**Methods:**

We analyzed a nationally representative sample of AIAN and White adults from the NHIS 2023 dataset. Welfare receipt was assessed as the receipt of any public assistance or welfare payments from state or local welfare offices. Educational attainment was categorized into three levels: less than high school (reference), high school diploma to some college, and college degree or higher. Logistic regression models were used to assess the relationship between educational attainment and welfare receipt, with separate analyses for AIAN and White adults to evaluate differential effects.

**Results:**

Higher educational attainment (high school diploma to some college and college degree or higher) was associated with lower odds of welfare receipt across both groups. However, the protective effect of a college degree was significantly weaker for AIAN adults compared to White adults. Consequently, AIAN adults remain at a higher risk of welfare reliance even with higher education, consistent with the Minorities’ Diminished Returns (MDRs) framework.

**Conclusions:**

Although educational attainment generally reduces welfare reliance, this protection is less pronounced for AIAN adults than for White adults. This discrepancy suggests that structural factors, segregation, and social stratification may undermine the economic and health benefits of education for racialized groups in the U.S. Addressing these disparities requires policy interventions that extend beyond education, emphasizing quality job opportunities, healthcare access, and reduced labor market discrimination for individuals with advanced educational credentials, regardless of race.

## 1. Introduction

Communities across the United States strive to reduce both overall welfare reliance and disparities in welfare receipt [1,2]. Welfare support programs, though essential for those in economic need, are often viewed as temporary assistance intended to bridge individuals toward greater economic stability [3–6]. High levels of welfare reliance can indicate structural inequities and limited economic opportunities, while disparities in welfare receipt highlight the uneven access to and benefits from socioeconomic resources across racial and ethnic groups [7]. Reducing both the overall dependence on welfare and the disparities within welfare receipt is essential not only for alleviating poverty but also for promoting equity and economic resilience within marginalized communities [8].

Educational attainment is widely recognized as a critical factor in reducing welfare receipt and reliance [9,10]. Higher education is typically associated with increased access to better job opportunities, higher income, and greater economic stability, all of which reduce the likelihood of welfare reliance [11–16]. For many, education serves as a pathway to financial independence and resilience, diminishing the need for public assistance [17]. However, while educational attainment is generally protective against welfare reliance, this protective effect is not equally distributed across all racial and ethnic groups.

The Minorities’ Diminished Returns (MDRs) framework [18] suggests that education and other socioeconomic resources may yield fewer protective benefits for racialized and minoritized populations compared to their White counterparts. This phenomenon, observed across various health and economic outcomes, reflects structural barriers and systemic inequities that limit the socioeconomic returns of education for marginalized groups. Consequently, despite similar educational achievements, minoritized populations may continue to face higher levels of economic insecurity and welfare reliance [19].

Much of the existing MDRs research has focused on Black [18,20–26] and Latino [27–30] populations, with limited investigation into American Indian/Alaska Native (AIAN) communities [31–33]. AIAN individuals represent a unique racial and cultural group with distinct historical, social, and economic challenges, including the legacy of colonization, forced relocation, and ongoing marginalization [34,35]. These factors may further affect how educational attainment influences welfare reliance within this population, yet little is known about the specific dynamics at play for AIAN adults [31–33]. Understanding the role of education in shaping welfare reliance for AIAN individuals, relative to White adults, is crucial for addressing disparities and informing targeted policy interventions.

The AIAN population in the United States has a unique and complex historical experience that shapes its current socioeconomic landscape [36–38]. Unlike other racial and ethnic groups, AIAN communities have endured centuries of colonization, forced relocation, cultural suppression, and systematic dispossession of land and resources, which has deeply impacted their social and economic trajectories [39–45]. Many AIAN individuals reside in geographically isolated areas or reservations, where limited access to quality education, healthcare, and employment opportunities persists [46–49]. Additionally, AIAN communities have often been subject to unique policies that have oscillated between forced assimilation and neglect, contributing to ongoing cycles of economic hardship and marginalization [50–52]. These historical and structural factors continue to influence the contemporary experiences of AIAN individuals, often limiting the socioeconomic benefits of resources such as education and reinforcing disparities in income, employment, and welfare reliance. Consequently, the AIAN population represents a distinct group for whom the pathways to economic security, even through higher education, may diverge significantly from those of other racial groups [53–56].

This study aimed to examine (1) the overall association between educational attainment and welfare receipt among U.S. adults and (2) whether the protective effects of education on welfare reliance differ between AIAN and White adults. We hypothesized that (1) higher educational attainment would generally be associated with lower welfare receipt among adults in the U.S. and (2) the protective effect of educational attainment on welfare receipt would be weaker for AIAN adults than for White adults, consistent with the MDRs framework. This analysis offers new insights into the impact of education on welfare reliance for AIAN communities, highlighting potential racial variations in the returns on education within the context of welfare reliance.

## 2. Methods

### 2.1. National Health Interview Survey (NHIS)

The National Health Interview Survey (NHIS) is the principal source of health data for the civilian, noninstitutionalized U.S. population, conducted by the National Center for Health Statistics (NCHS) since 1957. Authorized by the National Health Survey Act of 1956, NHIS continuously gathers data on health and disability across various demographics and socioeconomic groups, supporting research and policy initiatives within the Department of Health and Human Services and beyond [57].

### 2.2. NHIS 2023

In 2023, the NHIS conducted 29,522 Sample Adult interviews and 7,692 Sample Child interviews, achieving response rates of 47.0% and 44.9%, respectively. Data was collected through both in-person and telephone interviews, with 54.5% of interviews conducted at least partially by phone, similar to 2022 but higher than pre-pandemic levels in 2019 [58].

### 2.3. Design

NHIS is a cross-sectional, continuous survey covering the U.S. civilian noninstitutionalized population in the 50 states and the District of Columbia. Data was collected throughout the year, allowing monthly samples to be nationally representative. To manage costs and logistics, NHIS employs a geographically clustered sampling design [58].

### 2.4. Sampling

The sampling process begins by partitioning the United States into 1,689 geographic areas, based on counties or groups of counties. These areas are further stratified by population density in some states, while smaller states and the District of Columbia remain unstratified. Clusters within each stratum are selected proportionally, ensuring a representative national sample [58].

### 2.5. Sample

The NHIS targets individuals in households and noninstitutional group quarters, such as homeless shelters and group homes, but excludes active-duty military personnel, persons in long-term care institutions, and residents in correctional facilities [58].

### 2.6. Process and Interview

The U.S. Census Bureau conducts NHIS interviews under contract, deploying approximately 864 trained interviewers nationwide. Interviewers use computer-assisted personal interviewing (CAPI) technology, which facilitates question routing, real-time data entry, and data validation. Respondents receive an advance letter explaining NHIS participation, ensuring informed, voluntary consent. Interviews are conducted primarily in person, with follow-ups or special requests handled by phone [58].

### 2.7. Analytical Sample for this Paper

This paper used a sample of 19,829 participants, representative of 162,262,055 non-Latino White and AIAN adults in the U.S. Eligibility criteria included having data on race/ethnicity, identifying as non-Latino White or non-Latino AIAN, and being an adult. Individuals of any other race, as well as all Latino or Hispanic individuals, were excluded from the analysis.

### 2.8. Measures

#### Educational Attainment:

Participants reported their highest level of education as one of the following categories: 00 (Never attended/kindergarten only), 01 (Grade 1–11), 02 (12th grade, no diploma), 03 (GED or equivalent), 04 (High school graduate), 05 (Some college, no degree), 06 (Associate degree: occupational, technical, or vocational program), 07 (Associate degree: academic program), 08 (Bachelor’s degree, e.g., BA, BS, BBA), 09 (Master’s degree, e.g., MA, MS, MBA), and 10 (Professional or doctoral degree, e.g., MD, JD, PhD). For analysis, we consolidated these categories into three groups: (1) Less than high school diploma (categories 0–3), (2) Some college (categories 4–7), and (3) College degree or higher (categories 8+). Educational attainment was treated as a three-level categorical variable, with “less than high school diploma” as the reference category, and “some college” and “college degree or higher” as the other two levels compared to this reference.

### 2.9. Outcomes

#### Welfare Receipt (No=0, Yes=1):

All participants were asked if they receive any public assistance or welfare payments from a state or local welfare office, with options of “yes,” “no,” or “refused to answer.” This variable was coded as a binary outcome: 0 for “no” and 1 for “yes.”

#### Covariates:

Gender (female=0, male =1), age (years), marital status (other =0, married =1), last week employment status (other = 0, employed =1). All covariates were self-report.

### 2.10. Ethics

This study was conducted in compliance with ethical standards, ensuring the protection and confidentiality of participant information. All data were collected anonymously, and no identifying information was retained. The study adhered to the ethical principles outlined in the Declaration of Helsinki, emphasizing respect, beneficence, and justice in research practices. Written informed consent was obtained from all participants prior to their involvement in the study, following a clear explanation of the study’s purpose, procedures, potential risks, and benefits. Institutional Review Board (IRB) approval was obtained for NHIS to ensure that all ethical guidelines were rigorously followed throughout the research process. Current analysis used fully deidentified existing data and did not need a full IRB review.

### 2.11. Statistical Analysis

We conducted all analyses using Stata, accounting for the survey design variables, including survey weights and strata, to ensure accurate representation of the non-Latino White and non-Latino AIAN U.S. adult populations. Given our focus on these groups exclusively, we applied subpopulation logistic regression techniques. With four outcomes of interest, we initially ran four separate logistic regression models without interaction terms. These models tested the additive effects of race and education on each outcome, adjusting for age, gender, marital status, and employment status as covariates. As all participants were non-Latino, ethnicity was not included as a control variable. Next, we conducted a second set of four logistic regression models, this time including interaction terms between race and education. These models retained all covariates from the initial models, allowing us to assess whether the effect of education on each outcome differed between non-Latino White and AIAN adults. From each logistic regression model, we reported the odds ratios (OR), standard errors (SE), 95% confidence intervals (CI), and p-values. The results consistently showed that higher educational attainment was inversely associated with reliance on Social Security and disability-related income (odds ratios less than one), indicating a protective effect of education. However, the race-by-education interaction terms revealed that this protective effect was significantly weaker for AIAN adults compared to White adults, as evidenced by interaction odds ratios greater than one. This suggests a diminished return on education for AIAN Americans in terms of reducing reliance on Social Security and disability income sources. All results presented are representative of non-Latino White and AIAN adults.

## 3. Results

### 3.1. Descriptive Results

19,829 participants entered the analysis. Table 1 presents the descriptive statistics of the study sample, both overall and stratified by race. The overall sample consists predominantly of White individuals, comprising 97.99% (SE = 0.2527) of the sample, with AIAN individuals representing the remaining 2.01% (SE = 0.2527). The mean age of the participants was 50.45 years (SE = 0.19583). Educational attainment varied across participants, with 8.65% (SE = 0.30595) having less than a high school education, 54.93% (SE = 0.4946) being high school graduates, and 36.42% (SE = 0.54912) holding a bachelor’s degree or higher. In terms of marital status, 46.89% (SE = 0.48269) were categorized as “Other” (not married), while 53.11% (SE = 0.48269) reported being married. Regarding employment in the previous week, 43.23% (SE = 0.47056) were unemployed, while 56.77% (SE = 0.47056) were employed. Gender distribution was almost equal, with females making up 50.34% (SE = 0.41925) of the sample and males accounting for 49.66% (SE = 0.41925). Most participants (97.30%, SE = 0.16566) reported not receiving public assistance income, while 2.70% (SE = 0.16566) indicated that they did.

Table 1 also compares White and AIAN participants. In terms of educational attainment, AIAN individuals were more likely to have less than a high school education (15.13%, SE = 2.71735) compared to White participants (8.51%, SE = 0.3049). Furthermore, a smaller percentage of AIAN individuals had a bachelor’s degree or higher (19.24%, SE = 2.993) relative to White individuals (36.77%, SE = 0.55589), suggesting educational disparities between these groups. Marital status also differed, with a higher percentage of AIAN individuals identified as “Other” (not married) (62.44%, SE = 3.51207) compared to Whites (46.57%, SE = 0.47998). Conversely, the percentage of married participants was lower among AIAN individuals (37.56%, SE = 3.51207) than among White participants (53.43%, SE = 0.47998). In terms of employment status, a similar proportion of AIAN (44.84%, SE = 2.78464) and White (43.20%, SE = 0.47033) participants reported being unemployed in the previous week, with slightly more AIAN individuals being unemployed. The employed rates for both groups were similar, with 56.80% (SE = 0.47033) of White and 55.16% (SE = 2.78464) of AIAN participants employed. Gender distribution shows a higher percentage of females among AIAN individuals (60.70%, SE = 2.8748) compared to White individuals (50.12%, SE = 0.42006), whereas the proportion of males was higher among White participants (49.88%, SE = 0.42006) than among AIAN participants (39.30%, SE = 2.8748). When examining income from public assistance, a notable difference emerged, with a higher percentage of AIAN individuals (7.01%, SE = 1.37491) reporting reliance on public assistance compared to White individuals (2.61%, SE = 0.163). This suggests that AIAN individuals, despite similar employment rates, are more likely to receive public assistance, indicating potential economic instability. The mean age also varied between the two groups, with White participants having a higher average age (50.56 years, SE = 0.19592) than AIAN participants (45.38 years, SE = 1.1663).

### 3.2. Models 1 (Without Interaction)

Table 2 summarizes the results of logistic regression analyses examining factors associated with welfare receipt, without interaction terms. Race was a significant predictor, with AIAN individuals having over twice the odds of receiving welfare compared to non-AIAN individuals (OR = 2.02, SE = 0.43, 95% CI: 1.32 to 3.08, p = 0.001). Age was inversely associated with welfare receipt, with each additional year slightly reducing the odds (OR = 0.98, SE = 0.00, 95% CI: 0.98 to 0.99, p < 0.001).

Gender was also significant, with males having lower odds of welfare receipt than females (OR = 0.69, SE = 0.08, 95% CI: 0.56 to 0.87, p = 0.001). Family structure showed that married individuals had reduced odds of welfare receipt compared to non-married individuals (OR = 0.65, SE = 0.09, 95% CI: 0.50 to 0.84, p = 0.001). Employment in the previous week was strongly protective, as those employed had less than half the odds of welfare receipt compared to those unemployed (OR = 0.47, SE = 0.06, 95% CI: 0.37 to 0.60, p < 0.001).

Educational attainment was a significant factor, with individuals holding a high school diploma having lower odds of welfare receipt compared to those without a high school diploma (OR = 0.68, SE = 0.10, 95% CI: 0.50 to 0.91, p = 0.010). Those with a college degree or higher showed the greatest reduction in welfare reliance, with their odds significantly lower than those with less than a high school education (OR = 0.23, SE = 0.04, 95% CI: 0.16 to 0.33, p < 0.001).

### 3.3. Models 2 (With Interaction)

Table 3 presents the results of logistic regression analyses with interaction terms, examining how the association between educational attainment and welfare receipt differs by race. In this model, educational attainment showed a strong negative association with welfare receipt. Individuals with a high school diploma had lower odds of welfare reliance compared to those without a high school diploma (OR = 0.65, SE = 0.10, 95% CI: 0.48 to 0.88, p = 0.006), and individuals with a college degree or higher had even lower odds (OR = 0.21, SE = 0.04, 95% CI: 0.14 to 0.31, p < 0.001). The main effect of race (AIAN) was not significant (OR = 0.99, SE = 0.46, 95% CI: 0.39 to 2.48, p = 0.981), suggesting that, when interactions are included, the direct association between AIAN race and welfare receipt is not statistically significant. For individuals with a high school diploma, the interaction with AIAN race was not significant (OR = 2.13, SE = 1.14, 95% CI: 0.75 to 6.07, p = 0.155), indicating no significant difference in welfare receipt between AIAN and White individuals at this level of education. However, the interaction between having a college degree and AIAN race was significant (OR = 5.41, SE = 4.29, 95% CI: 1.14 to 25.65, p = 0.034), suggesting that AIAN individuals with a college degree had significantly higher odds of welfare reliance compared to their White counterparts with similar education levels.

Age remained inversely associated with welfare receipt, with each additional year reducing the odds (OR = 0.98, SE = 0.00, 95% CI: 0.98 to 0.99, p < 0.001).

Gender was a significant factor, with males showing lower odds of welfare receipt compared to females (OR = 0.69, SE = 0.08, 95% CI: 0.56 to 0.86, p = 0.001). Similarly, marital status was associated with welfare receipt, as married individuals had lower odds of receiving welfare than those who were not married (OR = 0.65, SE = 0.09, 95% CI: 0.50 to 0.84, p = 0.001). Employment status in the previous week continued to be protective, with employed individuals having significantly lower odds of welfare reliance compared to unemployed individuals (OR = 0.47, SE = 0.06, 95% CI: 0.37 to 0.60, p < 0.001).

## 4. Discussion

This study aimed to investigate (1) the association between educational attainment and welfare receipt among AIAN and White adults in the United States, and (2) whether the protective effects of educational attainment on welfare reliance differ between AIAN and White adults, consistent with the Minorities’ Diminished Returns (MDRs) framework. We hypothesized that (1) higher educational attainment would generally be associated with lower welfare receipt among adults in the U.S., and (2) the protective effect of higher educational attainment on welfare receipt would be weaker for AIAN adults than for White adults. These hypotheses were tested using data from the NHIS 2023.

This study contributes to the growing body of literature on Minorities’ Diminished Returns (MDRs) by revealing that the protective effect of educational attainment on welfare reliance is significantly weaker for AIAN adults compared to White adults. Although educational attainment generally correlates with lower welfare receipt, AIAN adults appear to experience less economic security from their educational achievements, consistent with the MDRs framework. These findings underscore the role of structural and systemic barriers in limiting the socioeconomic benefits typically associated with higher education for AIAN individuals, perpetuating economic inequities even among those with high educational credentials.

The first aim confirmed that higher educational attainment was generally associated with lower welfare reliance across both AIAN and White adults, supporting the notion that education provides economic advantages that typically reduce the need for welfare support. However, it is important to recognize that while educational attainment often correlates with decreased reliance on welfare, this finding should be interpreted with caution given the complexities surrounding how socioeconomic resources translate into actual economic stability. For both groups, higher education likely opens pathways to better employment and increased income, which typically reduce welfare reliance.

The second aim highlighted a pronounced difference in the protective effect of education between AIAN and White adults, with the association between higher education and reduced welfare receipt being notably weaker for AIAN adults. This finding aligns with the MDRs framework [18–27], suggesting that AIAN individuals do not experience the same economic benefits from education as their White counterparts. This diminished return may stem from structural and systemic factors, such as discrimination, geographic barriers, and limited access to high-quality job opportunities, which reduce the potential economic security that typically accompanies higher education. Consequently, despite comparable educational levels, AIAN adults remain more vulnerable to welfare reliance, underscoring the need for policies that address structural barriers beyond educational access.

### 4.1. Interpretation of Findings

The results of this study highlight a persistent disparity: while higher education tends to reduce welfare reliance, AIAN individuals with similar levels of education as their White counterparts remain disproportionately reliant on welfare. This aligns with MDRs theory, which posits that minoritized populations experience reduced socioeconomic returns from education and other resources. For AIAN adults, educational attainment does not appear to translate into economic stability to the same extent as it does for White adults, suggesting that additional, entrenched structural barriers may limit the opportunities and financial resilience of educated AIAN individuals.

### 4.2. Mechanisms and Structural Factors

There are several possible explanations for these diminished returns of education among AIAN adults. Structural racism, historical marginalization, and geographic segregation may all play a role in limiting the employment and income opportunities available to AIAN people, even those with higher education. AIAN individuals may face higher rates of discrimination in the labor market, more limited access to high-quality job opportunities, and less robust social networks in professional sectors, all of which can constrain the economic advantages that typically accompany educational attainment.

Moreover, residential and geographic factors unique to AIAN communities, including the prevalence of rural or reservation-based living, may restrict access to employment opportunities and limit access to healthcare, further diminishing the potential returns of education. These factors suggest that the economic and social environment in which AIAN individuals live plays a crucial role in shaping the outcomes of educational investment.

### 4.3. Policy Implications

The findings underscore the importance of developing targeted policies that go beyond merely increasing educational attainment among AIAN populations. While education is an important determinant of economic mobility, the MDRs framework suggests that, for AIAN individuals, structural reforms are essential to achieving equitable economic outcomes. Policymakers should consider interventions aimed at increasing job quality, ensuring equitable access to healthcare, and reducing discrimination in the labor market. Additionally, creating economic opportunities within AIAN communities, particularly in rural and reservation areas, could help address some of the structural limitations that contribute to these diminished returns.

Policies promoting workforce development, anti-discrimination measures in hiring and promotion, and targeted investment in AIAN communities could help close the gap in economic returns to education. Further, tailored support programs that address the unique challenges faced by educated AIAN adults, such as job placement services and workforce integration, may also alleviate the need for welfare reliance despite higher education.

### 4.4. Limitations

While this study provides valuable insights, certain limitations should be considered. First, the NHIS dataset captures welfare reliance and educational attainment at a single time point, limiting our ability to track changes in welfare reliance over time. Second, the study does not account for regional variations in economic conditions, which may impact welfare reliance among AIAN adults. Due to the low sample size of the AIAN participants, the coefficients had large confidence intervals. Future studies could enhance our understanding of MDRs among AIAN populations by employing longitudinal designs and incorporating regional economic variables to capture a more comprehensive picture of the structural factors at play.

## 5. Conclusion

In summary, this study demonstrates that while educational attainment reduces welfare reliance for both AIAN and White adults, the effect is less pronounced for AIAN individuals, revealing a critical disparity consistent with the Minorities’ Diminished Returns framework. Structural inequities appear to diminish the economic benefits of education for AIAN adults, underscoring the need for policies that address not only educational access but also systemic factors that inhibit economic stability among minoritized populations. These findings call for a multi-faceted approach that combines educational investment with targeted structural interventions to reduce welfare reliance and promote economic equity for AIAN communities.

## Figures and Tables

**Table 1.** Descriptives Overall and by Race

	All		White		AIAN	
	%	SE	%	SE	%	SE
Race						
White	97.99	0.25				
AIAN	2.01	0.25				
Education*						
Less than High School	8.64	0.30	8.51	0.30	15.12	2.71
High School Graduate	54.93	0.49	54.71	0.50	65.629	2.44
Bachelor or More	36.42	0.54	36.77	0.55	19.24	2.99
Marital Status*						
Other	46.88	0.482	46.56	0.47	62.43	3.51
Married	53.11	0.482	53.43	0.47	37.56	3.51
Last Week Employment*						
Unemployed	43.23	0.47	43.19	0.47	44.83	2.78
Employed	56.76	0.47	56.80	0.47	55.16	2.78
Gender*						
Female	50.33	0.41	50.12	0.42	60.70	2.87
Male	49.66	0.41	49.87	0.42	39.29	2.87
Income From Public Assistance*						
No	97.29	0.16	97.38	0.16	92.99	1.37
Yes	2.70	0.16	2.61	0.16	7.00	1.37
	Mean	SE	Mean	SE	Mean	SE
Age (Years)*	50.45	.195	50.55	.19	45.37	1.16

Data Source: National Center for Health Statistics, National Health Interview Survey, 2023.

* p < 0.05

**Table 2.** Summary of logistic regressions without interactions

	Odds ratio	Linearized Std. Err.	[95% conf.	interval]	p
Race (AIAN)	2.02	0.43	1.32	3.08	0.001
Age (Years)	0.98	0.00	0.98	0.99	< 0.001
Gender (Male)	0.69	0.08	0.56	0.87	0.001
Family Structure (Married)	0.65	0.09	0.50	0.84	0.001
Employment (Employed During Last Week)	0.47	0.06	0.37	0.60	< 0.001
Educational Attainment					
Less than High School Diploma	Ref.				
High School Diploma	0.68	0.10	0.50	0.91	0.010
College Degree or More	0.23	0.04	0.16	0.33	< 0.001
Intercept	0.21	0.05	0.14	0.33	< 0.001

Data Source: National Center for Health Statistics, National Health Interview Survey, 2023.

**Table 3.** Summary of logistic regressions with interactions

	Odds ratio	Linearized Std. Err.	[95% conf.	interval]	p
Race (AIAN)	0.99	0.46	0.39	2.48	0.981
Age (Years)	0.98	0.00	0.98	0.99	< 0.001
Gender (Male)	0.69	0.08	0.56	0.86	0.001
Family Structure (Married)	0.65	0.09	0.50	0.84	0.001
Employment (Employed During Last Week)	0.47	0.06	0.37	0.60	< 0.001
Educational Attainment					
Less than High School Diploma					
High School Diploma	0.65	0.10	0.48	0.88	0.006
College Degree or More	0.21	0.04	0.14	0.31	< 0.001
Education × Race					
High School Diploma × Race	2.13	1.14	0.75	6.07	0.155
College Degree or More × Race	5.41	4.29	1.14	25.65	0.034
Intercept	0.22	0.05	0.15	0.34	< 0.001

Data Source: National Center for Health Statistics, National Health Interview Survey, 2023.

## References

[1] Blau J, Abramovitz M: The dynamics of social welfare policy. Oxford University Press, USA, 2010.

[2] Cantillon B, Van Mechelen N, Pintelon O, Van den Heede A: Social redistribution, poverty and the adequacy of social protection. Reconciling work and poverty reduction: how successful are European welfare states. 2014:157–184.

[3] Lansdale RT: Public Welfare and Self-Reliance. The American Journal of Economics and Sociology. 1949, 9:71–76.

[4] Marsland D: Self Reliance. Transaction Publishers, 1995.

[5] Dahler AM, Petersen LH, Andersen PT: Implementing welfare technologies: On wash toilets and self-reliant citizens. STS Encounters. 2018, 10.

[6] Ziliak ST: Self-reliance before the welfare state: evidence from the charity organization movement in the United States. The Journal of Economic History. 2004, 64:433–461.

[7] Scott EK, London AS, Gross G: “i try not to depend on anyone but me”: welfare‐reliant women’s perspectives on self‐sufficiency, work, and marriage. Sociological Inquiry. 2007, 77:601–625.

[8] Takada S: The relationship between education and child welfare in Japanese children’s self-reliance support facilities. Contemporary Japan. 2018, 30:60–77.

[9] Duncan GJ, Hill MS, Hoffman SD: Welfare dependence within and across generations. Science. 1988, 239:467–471. 3277267 10.1126/science.3277267

[10] Barrett GF: The effect of educational attainment on welfare dependence: Evidence from Canada. Journal of Public Economics. 2000, 77:209–232.

[11] Ross CE, Mirowsky J: Does employment affect health? J Health Soc Behav. 1995, 36:230–243. 7594356

[12] Ross CE, Mirowsky J: Refining the association between education and health: the effects of quantity, credential, and selectivity. Demography. 1999, 36:445–460. 10604074

[13] Ross CE, Mirowsky J: Disorder and decay: The concept and measurement of perceived neighborhood disorder. Urban Affairs Review. 1999, 34:412–432.

[14] Ross CE, Mirowsky J: Refining the association between education and health: the effects of quantity, credential, and selectivity. Demography. 1999, 36:445–460. 10604074

[15] Ross CE, Wu C-l: The links between education and health. American sociological review. 1995:719–745.

[16] Ross CE, Wu C-L: Education, age, and the cumulative advantage in health. Journal of health and social behavior. 1996:104–120. 8820314

[17] Beaulieu N, Duclos J-Y, Fortin B, Rouleau M: Intergenerational reliance on social assistance: Evidence from Canada. Journal of Population Economics. 2005, 18:539–562.

[18] Assari S: Health disparities due to diminished return among black Americans: Public policy solutions. Social Issues and Policy Review. 2018, 12:112–145.

[19] Assari S: Unequal Gain of Equal Resources across Racial Groups. Int J Health Policy Manag. 2018, 7:1–9. 10.15171/ijhpm.2017.90 29325397 PMC5745862

[20] Assari S: Diminished Economic Return of Socioeconomic Status for Black Families. Soc Sci (Basel). 2018, 7. 10.3390/socsci7050074 PMC7440031 32832108

[21] Assari S: Family Socioeconomic Position at Birth and School Bonding at Age 15; Blacks’ Diminished Returns. Behav Sci (Basel). 2019, 9. 10.3390/bs9030026 PMC6466592 30861987

[22] Assari S: Parental Educational Attainment and Academic Performance of American College Students; Blacks’ Diminished Returns. J Health Econ Dev. 2019, 1:21–31. 31372601 PMC6673665

[23] Assari S: American Children’s Screen Time: Diminished Returns of Household Income in Black Families. Information. 2020, 11:538. 33282415 10.3390/info11110538

[24] Assari S: Blacks’ Diminished Health Returns of Educational Attainment: Health and Retirement Study. J Med Res Innov. 2020, 4. 10.32892/jmri.212 34966877

[25] Assari S, Boyce S, Bazargan M: Subjective Family Socioeconomic Status and Adolescents’ Attention: Blacks’ Diminished Returns. Children. 2020, 7:80. 32718077 10.3390/children7080080 PMC7464278

[26] Assari S, Caldwell CH, Mincy R: Family Socioeconomic Status at Birth and Youth Impulsivity at Age 15; Blacks’ Diminished Return. Children (Basel). 2018, 5. 10.3390/children5050058 PMC5977040 29724004

[27] Assari S: Minorities’ Diminished Returns of Educational Attainment on Life Satisfaction among Black and Latino Adults in the United States. Journal of Medicine, Surgery, and Public Health. 2024:100091. 10.1016/j.glmedi.2024.100091 PMC10900246 38425566

[28] Assari S: Diminished returns of educational attainment on life satisfaction among Black and Latino older adults transitioning into retirement. Journal of Medicine, Surgery, and Public Health. 2024, 2:100091. 10.1016/j.glmedi.2024.100091

[29] Assari S: Latinos’ diminished returns of educational attainment on reducing food insecurity: the role of ethnic disparities in family structure and employment. Front Public Health. 2024, 12:1407005. 10.3389/fpubh.2024.1407005 39224560 PMC11366641

[30] Assari S, Boyce S, Caldwell CH, Bazargan M: Parent Education and Future Transition to Cigarette Smoking: Latinos’ Diminished Returns. Front Pediatr. 2020, 8:457. 10.3389/fped.2020.00457 32974240 PMC7466764

[31] Assari S: American Indian, Alaska Native, Native Hawaiian, and Pacific Islander Children’s Body Mass Index: Diminished Returns of Parental Education and Family Income. Res Health Sci. 2020, 5:64–84. 10.22158/rhs.v5n1p64 34308086

[32] Assari S, Bazargan M: Protective Effects of Educational Attainment Against Cigarette Smoking; Diminished Returns of American Indians and Alaska Natives in the National Health Interview Survey. Int J Travel Med Glob Health. 2019, 7:105–110. 10.15171/ijtmgh.2019.22 31772950 PMC6879009

[33] Assari S, Zare H: Unequal Effect of Educational Attainment on Reducing Poverty and Welfare; Diminished Returns of American Indian/Alaska Native Populations. J Rehabil Ther. 2024, 6:1–11. 10.29245/2767-5122/2024/2.1143 PMC11296615 39100915

[34] Jaimes MA: The state of Native America: Genocide, colonization, and resistance. South End Press, 1992.

[35] Hodge DR, Limb GE, Cross TL: Moving from colonization toward balance and harmony: A Native American perspective on wellness. Social work. 2009, 54:211–219. 19530568 10.1093/sw/54.3.211

[36] van Breda A: Health issues facing Native American children. Pediatr Nurs. 1989, 15:575–577. 2616231

[37] Warren-Mears V, Ritchey J, Larson B, : Tribal Epidemiology Centers and Native American Health. J Acad Nutr Diet. 2016, 116:769–770. 10.1016/j.jand.2016.02.017 27126149

[38] Watson L: Sidebar: History Shaping the Future: How History Influences Health in North Carolina Native American Communities. N C Med J. 2021, 82:401–402. 10.18043/ncm.82.6.401 34750216

[39] Smith EM: Health care for native Americans: who will pay? Health Affairs. 1987, 6:123–128. 3556366 10.1377/hlthaff.6.1.123

[40] Struthers R, Baker M, Savik K: Cardiovascular risk factors among Native American women Inter-Tribal Heart Project participants. J Obstet Gynecol Neonatal Nurs. 2006, 35:482–490. 10.1111/j.1552-6909.2006.00069.x 16881992

[41] Struthers R, Lowe J: Nursing in the Native American culture and historical trauma. Issues Ment Health Nurs. 2003, 24:257–272. 10.1080/01612840305275 12623685

[42] Tan A, Fujioka Y, Lucht N: Native American stereotypes, TV portrayals, and personal contact. Journalism & Mass Communication Quarterly. 1997, 74:265–284.

[43] Tom-Orme L: Chronic disease and the social matrix: a native American diabetes intervention. Recent Adv Nurs. 1988, 22:89–109. 3231736

[44] Wescott S, Mittelstet B: Three Levels of Autonomy and One Long-Term Solution for Native American Health Care. AMA J Ethics. 2020, 22:E856–861. 10.1001/amajethics.2020.856 33103647

[45] Zephier Olson MD, Dombrowski K: A systematic review of Indian boarding schools and attachment in the context of substance use studies of Native Americans. Journal of racial and ethnic health disparities. 2020, 7:62–71. 31452147 10.1007/s40615-019-00634-4

[46] Allison M, Rivers PA, Fottler MD: Can Community Health Center funding enhance health services for Native American tribes and organizations? J Health Care Poor Underserved. 2004, 15:193–205. 10.1353/hpu.2004.0016 15253373

[47] Allison MT, Rivers PA, Fottler MD: Future public health delivery models for Native American tribes. Public Health. 2007, 121:296–307. 10.1016/j.puhe.2006.11.005 17289095

[48] Campos-Outcalt D, Ellis J, Aickin M, Valencia J, Wunsch M, Steele L: Prevalence of cardiovascular disease risk factors in a southwestern Native American tribe. Public Health Rep. 1995, 110:742–748. 8570829 PMC1381818

[49] Oddo VM, Walkinshaw LP, Jones-Smith JC: Casino Ownership and Health-Related Community Resources Among Native American Tribes in California. Prev Chronic Dis. 2019, 16:E14. 10.5888/pcd16.180252 30730831 PMC6395078

[50] Almas N, Mazhar S: The Cultural Assimilation of the Native Indians by the Colonizers in the United States of America with Special Reference to Their Languages. 2024.

[51] Beatty WW: The Goal of Indian Assimilation. Canadian Journal of Economics and Political Science/Revue canadienne de economiques et science politique. 1946, 12:395–404.

[52] Lee C: Civilizing Paternalism: Mill, Autonomy, and Indian Assimilation Policies. 2021.

[53] Braveheart-Jordan M, DeBruyn L: So she may walk in balance: Integrating the impact of historical trauma in the treatment of Native American Indian women. 1995.

[54] Evans-Campbell T: Historical trauma in American Indian/Native Alaska communities: a multilevel framework for exploring impacts on individuals, families, and communities. J Interpers Violence. 2008, 23:316–338. 10.1177/0886260507312290 18245571

[55] Jock BWI, Dana-Sacco G, Arscott J, : “We’ve Already Endured the Trauma, Who is Going to Either End that Cycle or Continue to Feed It?”: The Influence of Family and Legal Systems on Native American Women’s Intimate Partner Violence Experiences. J Interpers Violence. 2022, 37:NP20602–NP20629. 10.1177/08862605211063200 35114840 PMC9346087

[56] Tehee M, Buchwald D, Booth-LaForce C, Omidpanah A, Manson SM, Goins RT: Traumatic Stress, Social Support, and Health Among Older American Indians: The Native Elder Care Study. J Gerontol B Psychol Sci Soc Sci. 2019, 74:908–917. 10.1093/geronb/gbx163 29304244 PMC6566328

[57] Botman S, Moriarity CL: Design and estimation for the national health interview survey, 1995–2004. 2000. 11707926

[58] Statistics. NCfH: National Health Interview Survey, 2023 survey description. 2024.

